# Early determination of the dorsal-ventral axis in endochondral ossification in mice

**DOI:** 10.1093/jbmr/zjaf086

**Published:** 2025-06-23

**Authors:** Sixun Wu, Hirotaka Matsumoto, Jumpei Morita, Mina Yamabe, Azumi Noguchi, Shinsuke Ohba, Noriaki Ono, Yuki Matsushita

**Affiliations:** Department of Skeletal Development and Regenerative Biology, Nagasaki University Graduate School of Biomedical Sciences, Nagasaki 852-8588, Japan; Leading Medical Research Core Unit, Life Science Innovation, Nagasaki University Graduate School of Biomedical Sciences, Nagasaki 852-8523, Japan; School of Information and Data Sciences, Nagasaki University, Nagasaki 852-8521, Japan; Department of Skeletal Development and Regenerative Biology, Nagasaki University Graduate School of Biomedical Sciences, Nagasaki 852-8588, Japan; Department of Skeletal Development and Regenerative Biology, Nagasaki University Graduate School of Biomedical Sciences, Nagasaki 852-8588, Japan; Department of Skeletal Development and Regenerative Biology, Nagasaki University Graduate School of Biomedical Sciences, Nagasaki 852-8588, Japan; Leading Medical Research Core Unit, Life Science Innovation, Nagasaki University Graduate School of Biomedical Sciences, Nagasaki 852-8523, Japan; Department of Tissue and Developmental Biology, Graduate School of Dentistry, Osaka University, Osaka 565-0871, Japan; Department of Diagnostic and Biomedical Sciences, University of Texas Health Science Center at Houston School of Dentistry, Houston, TX 77054, United States; Department of Skeletal Development and Regenerative Biology, Nagasaki University Graduate School of Biomedical Sciences, Nagasaki 852-8588, Japan; Leading Medical Research Core Unit, Life Science Innovation, Nagasaki University Graduate School of Biomedical Sciences, Nagasaki 852-8523, Japan

**Keywords:** bone development, endochondral ossification, mesenchymal condensation, cartilage anlage, dorsal-ventral axis, dorsoventral axis, lineage tracing, *Fgfr3*, *Dlx5*, skeletal progenitor cells

## Abstract

Endochondral ossification is a highly coordinated process involving distinct progenitor cell populations within the mesenchymal condensation and subsequent cartilage anlage and perichondrium, all of which drive skeletal formation. Cell-type specific lineage tracing conducted to understand fetal bone development has revealed various fates of early skeletal cells. However, the underlying continuous and precise cellular dynamics of fetal skeletal cells, particularly along the dorsoventral axis, remain unclear. Here, we show that spatiotemporally specific skeletal progenitor cells in the early developmental stage contribute to the dorsal-ventral axis in a manner that is strictly determined during initial developmental stages. Lineage-tracing experiments using *Fgfr3-creER* and *Dlx5-creER* lines revealed that *Fgfr3*^+^ cells in mesenchymal condensation exclusively contributed to hypertrophic chondrocytes and the dorsal side of the resting and proliferating zones within the cartilage anlage. These cells made dorsal-restricted contributions to skeletal development, including growth plate chondrocytes, trabecular and cortical osteoblasts, and bone marrow stromal cells. Functional ablation of *Fgfr3*^+^ cells using the *Rosa26*^iDTA^ (inducible diphtheria toxin fragment A) allele during the mesenchymal condensation stage caused severe disruption in long-bone development, underscoring its indispensable role in initiating skeletal growth. Collectively, these findings suggest that the condensation stage is pivotal for the formation of skeletal progenitors and dorsoventral patterning during bone development. Understanding these mechanisms will provide insight into skeletal growth disorders and therapeutic strategies for bone regeneration.

## Graphical Abstract

**Graphical Abstract:**
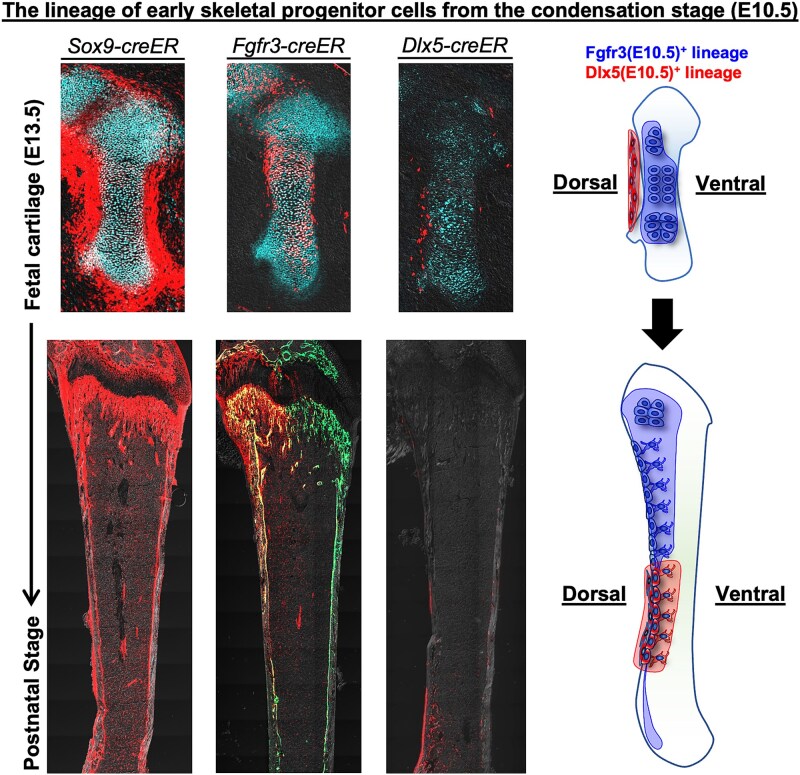

## Introduction

Mesenchymal condensation is a critical initial step in the formation of skeletal structures during early development of long bones. At E10.5-E11.5 in mice, expression of key regulatory transcription factors induce undifferentiated mesenchymal cells to accumulate in regions destined for bone development.^1^  *Prrx1* and *Sox9* play key roles in this process, with *Prrx1* labeling the early limb bud mesenchyme^2^ and *Sox9* serving as a major regulator of chondrogenesis, which is essential for initiating cartilage formation.^3^^,^^4^  *Sox9* expression promotes chondrocyte differentiation and cartilage matrix deposition, setting the foundation for subsequent endochondral ossification.^5–7^

Following mesenchymal condensation, cartilage anlage is established at E12.5-13.5. At this stage, 2 structurally distinct tissues and cell populations, classified as chondrocytes and perichondrial cells, are present.^8^ A recent single-cell RNA sequencing (scRNA-seq) analysis revealed the heterogeneity of these cells, identifying 2 distinct skeletal origins: populations of FGF receptor type 3 (*Fgfr3*)-expressing chondrocytes and *Dlx5*-expressing perichondrial cells.^9^  *Fgfr3* is a transmembrane receptor tyrosine kinase essential for regulating cell growth and differentiation during skeletal development, with mutations associated with disorders such as achondroplasia.^10^^,^^11^  *Dlx5* is a homeobox transcription factor essential for skeletal development and the differentiation of osteoblasts and chondrocytes.^12^ Histologically, *Fgfr3*^+^ chondrocytes are located in the center of the entire *Sox9*^+^ cartilage anlage. These *Fgfr3*^+^ chondrocytes contribute to most of the skeletal elements in the neonatal stage and predominantly to the development of the metaphyseal skeleton, mapping another key developmental pathway.^13^ In contrast, the perichondrium is divided into the inner *Sp7*/*Osx*^+^^14^ and outer *Dlx5*^+^ cell layers. These 2 types of perichondrial cells contribute to bone development until early postnatal stages. Remarkably, the osteogenic potential of *Osx*^+^ perichondrial cells is transient, indicating their distinct and temporal contribution to bone formation.^15^ Recent lineage-tracing studies highlighted that *Dlx5*^+^ cells in the outer perichondrium contribute permanently to skeletal elements, particularly in the diaphyseal region.^9^ In the postnatal stage, bone marrow stromal cells derived from the *Fgfr3*^+^ cartilage anlage and those originating from the outer *Dlx5*^+^ perichondrium within the bone marrow exhibit distinct characteristics. Anlage-derived *Fgfr3*^+^ stromal cells exhibit osteoblastic and chondrogenic properties, whereas outer *Dlx5*^+^ perichondrium-derived cells display adipocyte-like features.^9^ This discovery revealed that cells forming adult bone are precisely programmed from these 2 distinct origins during embryonic development, and their fates have already been determined.

To date, the cellular origins and comprehensive landscape of cellular dynamics in postnatal and adult stages have been elucidated by partitioning the perichondrium into inner and outer layers during developmental stages. Notably, the cartilage anlage, contrary to the perichondrium, contributes substantially to the postnatal skeleton and is structurally organized into distinct zones—namely, resting, proliferating, and hypertrophic—each of which plays a unique role in the longitudinal growth of bones.^16–18^

However, specific cells within the cartilage anlage that contribute to the adult skeleton and their underlying mechanism remain unclear. Elucidating the contributions of developmental stage-cell localization to specific cells postnatally and during growth, along with cell-specific gene expression and characteristics, could potentially lead to a comprehensive understanding of skeletal disorders associated with development and growth and facilitate the development of therapeutic interventions.

In this study, we focused on cells that specifically labeled subsets of mesenchymal condensation and cartilage anlages. By elucidating their lineages and functions through mouse genetic tools, we sought to reveal skeletal cellular dynamics with potential implications for understanding human biology in the future.

## Materials and methods

### Mouse strains

*Sox9-creER* mouse has been described previously,^3^  *Fgfr3-creER* (JAX025809), *Dlx5-creER* (JAX010705), *Rosa26-CAG-loxP-stop-loxP-tdTomato* (Ai14: *R26R-tdTomato*, JAX007914), *Col1a1(2.3 kb)-GFP* (JAX013134), *Rosa26-SA-loxP-GFP-stop-loxP-DTA* (JAX006331), *FVB/NJ* (JAX001800), and *C57BL/6 J* (JAX000664) mice were acquired from the Jackson laboratory. All experiments were performed in accordance with the protocols approved by the Animal Care and Use Committee of Nagasaki University (#2204261788 and #2404301942) and the University of Texas Health Science Center at Houston’s Animal Welfare Committee (#AWC-21-0070). All mice were housed in a specific pathogen-free condition, and analyzed in a mixed or the C57BL/6 J background. Mice were housed in static microisolator cages with ad libitum access to water and food. Animal rooms were climate-controlled to provide temperatures of 22-23 °C and 40%-65% humidity on a 12-h light/dark cycle. For all breeding experiments, *creER* transgenes were maintained in male breeders to avoid spontaneous germline recombination. Mice were identified by micro-tattooing or ear tags. Tail biopsies of mice were lysed by a HotShot protocol (incubating the tail sample at 95 °C for 1 h in an alkaline lysis reagent followed by neutralization) and used for PCR-based genotyping (DreamTaq Green PCR Master Mix, Thermo Fisher K1082). Perinatal mice were also genotyped fluorescently (Handy light, OptoCode LEDGFP-3WOF and LED530-3WRF) whenever possible. Mice were euthanized by over-dosage of carbon dioxide or decapitation under inhalation anesthesia in a drop jar (Isoflurane, Fujifilm 099-06571).

### Tamoxifen and induction of cre-loxP recombination

Tamoxifen (CAY 13258) was dissolved entirely in 100% ethanol. Subsequently, a proper volume of sunflower seed oil (Wako DLJ6115) was added to the tamoxifen-ethanol mixture and rigorously mixed. The tamoxifen-ethanol-oil mixture was incubated at 50 °C in a chemical hood until the ethanol evaporated completely. The tamoxifen-oil mixture was stored at room temperature until use. Tamoxifen was injected at a dose of 3 mg into pregnant mice i.p. using a 26-1/2-gauge needle (Terumo NN-2613S).

### Computational analysis for scRNA-seq

Single-cell RNAseq dataset (GSE144411 and GSE126966) were downloaded from the National Center for Biotechnology Information (NCBI)’s Gene Expression Omnibus (GEO) database. Downstream analysis was performed using the Seurat R package (v.5.1).^19^ We filtered out cells with less than 2000 genes per cell and more than 20% mitochondrial read content. Then, we applied the resolution of the curse of dimensionality (RECODE) algorithm^20^ to the Seurat object and used it as the default noise reduction method (applied only to GSE144411). The downstream analysis steps include normalization (LogNormalize), identification of highly variable genes across the single cells (vst method), scaling with default settings, dimensionality reduction using principal component analysis (PCA) and uniform manifold approximation and projection (UMAP), unsupervised clustering, and the discovery of differentially expressed cell-type specific markers.

### Histology and immunohistochemistry

Samples were dissected and fixed in 4% paraformaldehyde for a proper period, ranging from 3 h to overnight at 4 °C, then decalcified in 15% EDTA for a proper period, typically 7 to 14 d for postnatal samples. Embryonic samples were not decalcified. Subsequently, samples were cryoprotected in 30% sucrose/PBS solutions and then in 30% sucrose/PBS:OCT (1:1) solutions, each at least overnight at 4 °C. Samples were embedded in an OCT compound (Tissue-Tek, Sakura) and transferred on a sheet of dry ice to solidify the compound. Samples were cryosectioned at 10 μm using a cryostat (Leica CM1850) and adhered to positively charged glass slides (Matsunami MAS). Sections were postfixed in 4% paraformaldehyde for 10 min at room temperature. For immunostaining, sections were permeabilized with 0.25% TritonX/TBS for 10 min, blocked with 3% BSA/TBS for 30 min and incubated with rabbit anti-SOX9 polyclonal Ab (1:200, EMD-Millipore, AB5535) overnight at 4 °C, and subsequently with Alexa Fluor 633-conjugated goat anti-rabbit IgG (1:400, Invitrogen, A21071) for 2 h at room temperature. Sections were further incubated with DAPI (4′,6-diamidino-2-phenylindole, 5 μg/mL, CAY 14285) to stain nuclei prior to imaging. RNAscope in situ hybridization was performed with RNAscope Multiplex Fluorescent Detection Kit v2 (Advanced Cell Diagnostics 323 100) using *Sox9* (401051) and *Fgfr3* (440771) probes according to the manufacturer’s protocol.

### Imaging and cell quantification

Images were captured by an automated inverted fluorescence microscope with a structured illumination system (Zeiss Axio Observer Z1 with ApoTome.3 system) and Zen 2 (blue edition) software. The filter settings used were: FL Filter Set 34 (Ex. 390/22, Em. 460/50 nm), Set 38 HE (Ex. 470/40, Em. 525/50 nm), Set 43 HE (Ex. 550/25, Em. 605/70 nm), Set 50 (Ex. 640/30, Em. 690/50 nm), and Set 63 HE (Ex. 572/25, Em. 629/62 nm). The objectives used were: Fluar 2.5×/0.12, EC Plan-Neofluar 5×/0.16, Plan-Apochromat 10×/0.45, EC Plan-Neofluar 20×/0.50, EC Plan-Neofluar 40×/0.75, and Plan-Apochromat 63×/1.40. Images were typically tile-scanned with a motorized stage, Z-stacked and reconstructed by a maximum intensity projection (MIP) function. Differential interference contrast (DIC) was used for objectives higher than 10×. Representative images of at least 4 independent biological samples are shown in the figures. Quantification of cells on sections was performed using NIH Image J software. For the quantification of the perichondrium, only DAPI^+^ cells located within 2 cell layers surrounding the SOX9^+^ cartilage were included. To quantify the selected region in cartilage anlage, we analyzed 3 defined regions: Area a (dorsal resting zone), Area b (ventral resting zone), and Area c (proliferative zone). Each area was measured as a 200 μm × 150 μm rectangle, ensuring uniform analysis. Area placement was determined based on anatomical landmarks within the cartilage anlage.

### Statistical analysis

Results are presented as mean values ± SD. Statistical evaluation was conducted by the 2-tailed Mann-Whitney’s *U* test, 2-tailed Wilcoxon matched-pairs signed rank test, 2-tailed One-way ANOVA followed by Dunnett’s multiple comparison test or Log-rank (Mantel-Cox) test using GraphPad Prism software. A *p* value of <.05 was considered significant. No statistical method was used to predetermine the sample size. The sample size was determined based on previous literature and our previous experience to give sufficient standard deviations of the mean so as not to miss a biologically important difference between groups. The experiments were not randomized. All of the mice with the desired genotypes were used for experiments. The investigators were not blinded during experiments and outcome assessments. One femur from each mouse was arbitrarily chosen for histological analysis. Genotypes were not particularly highlighted during quantification.

## Results

### Skeletal cell-related gene expression profiles in mesenchymal condensation

**Figure 1:**
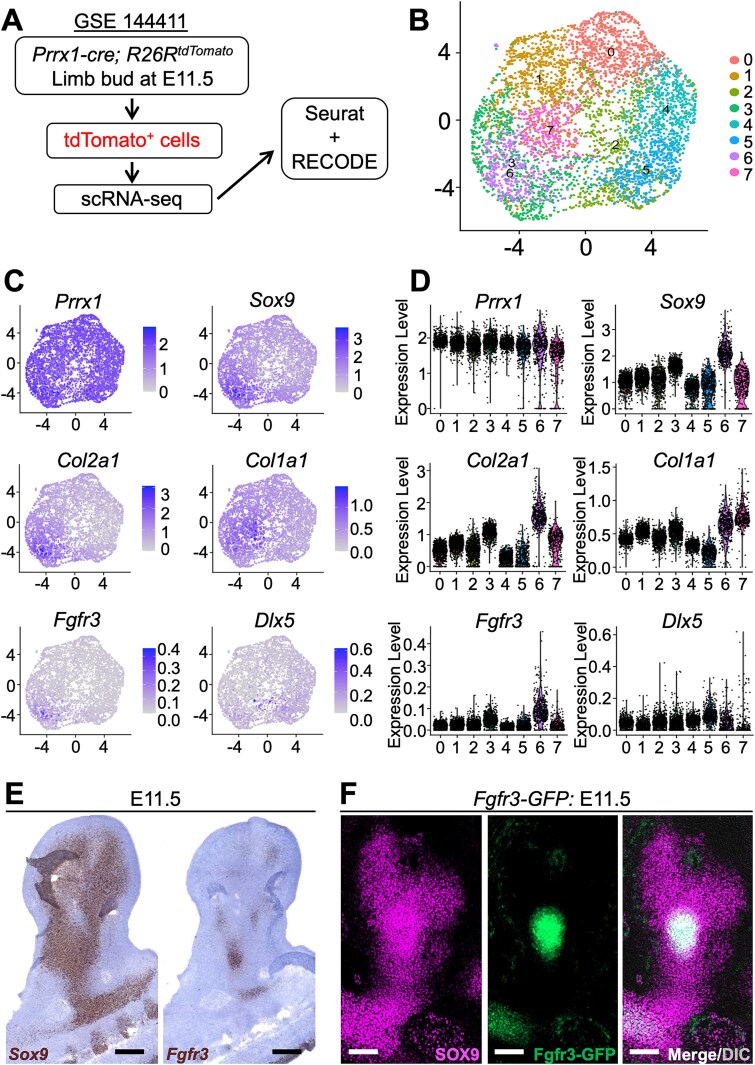
Skeletal cell-related gene expression profiles in mesenchymal condensation. (A) Schematic overview of the single-cell RNA sequencing analysis pipeline using the Seurat and resolution of the curse of dimensionality (RECODE) algorithms applied to the Gene Expression Omnibus dataset (GSE144411). FACS-isolated tdTomato^+^ cells were obtained from *Prrx1-cre*; *R26R*^tdTomato^ mouse limb buds at E11.5. (B) UMAP plot displaying 8 transcriptionally distinct clusters (Clusters 0-7). (C) Feature plots generated with RECODE showing the expression levels of key skeletal development-related genes (*Prrx1*, *Sox9*, *Col2a1*, *Col1a1*, *Fgfr3*, and *Dlx5*) across the clusters. Color intensity indicates normalized gene expression levels. (D) Violin plots demonstrating the expression distribution of the same representative genes across the identified clusters, emphasizing cluster-specific gene expression patterns. (E) RNAscope in situ detection of *Sox9* and *Fgfr3* (brown) in E11.5 limb bud. Scale bar: 200 μm. (F) Sox9 immunostaining in E11.5 limb bud of *Fgfr3-GFP* mice. Scale bar: 200 μm.

To investigate the gene expression landscape of mesenchymal condensation during early skeletal development, we downloaded the scRNA-seq dataset of tdTomato^+^ cells isolated from *Prrx1-cre*; *R26R*^tdTomato^ mouse limb buds at E11.5^21^ (Figure 1A) (GSE144411) from the NCBI GEO database. The dataset was processed using the Seurat^19^ and RECODE^20^ platforms for clustering and analysis. Using unsupervised clustering, we identified 8 distinct cell clusters, as shown in the UMAP plot (Figure 1B). These clusters represent transcriptionally heterogeneous populations within tdTomato^+^ mesenchymal cells, reflecting the diversity of cellular states during early skeletal development.

We focused on the expression of several key skeletal development-related genes, including *Prrx1*, *Sox9*, *Col2a1*, *Col1a1*, *Fgfr3*, and *Dlx5* (Figure 1C, D). *Prrx1* was broadly expressed across multiple clusters, and *Sox9* was strongly expressed, particularly in Clusters 3 and 6, both consistent with their roles in early mesenchymal condensation and chondrogenesis.^21^^,^^22^ These findings reaffirm their critical functions in initiating skeletal lineage. *Col2a1* expression corresponded to *Sox9* expression. It was explicitly enriched in Cluster 6, with moderate expression observed in Cluster 3. *Col1a1* was highly expressed in Cluster 7 and moderately expressed in Cluster 6. Notably, *Fgfr3* was exclusively expressed in Cluster 6, whereas *Dlx5* did not exhibit any specific expression pattern. Given their overall low expression levels, it is plausible that their expression is only beginning to be initiated at this stage of development.

To validate our single-cell transcriptomic findings within an anatomical context, we examined the histological expression patterns of the candidate markers at E11.5. RNAscope analysis revealed that *Sox9* mRNA was broadly expressed throughout the mesenchymal condensation, whereas *Fgfr3* mRNA was confined to a much smaller area within the *Sox9*^+^ domain (Figure 1E). Notably, this distinct spatial restriction recapitulates our scRNA-seq findings: *Sox9* is detected across multiple clusters, while *Fgfr3* expression is limited to Cluster 6. To further confirm this, we utilized *Fgfr3*-GFP reporter mice and performed immunohistostaining for SOX9 at E11.5 (Figure 1F). GFP fluorescence similarly delineated a tight central subpopulation embedded within the larger SOX9^+^ condensation. Taken together, these histological validations confirm that the 8 scRNA clusters correspond to anatomically distinct cell populations in the limb bud, and *Fgfr3*^+^ cells represent a molecularly and spatially defined subset within the early Sox9^+^ condensation, in precise concordance with their exclusive localization to Cluster 6.

These findings demonstrate the heterogeneity of mesenchymal cells during the condensation phase and provide insight into how specific gene-expression programs govern their differentiation into various skeletal lineages. The spatiotemporal regulation of these genes underscores their importance in the formation of distinct skeletal structures.

### Contribution of spatiotemporally specific embryonic Sox9-creER-targeted cells to long-bone development

**Figure 2:**
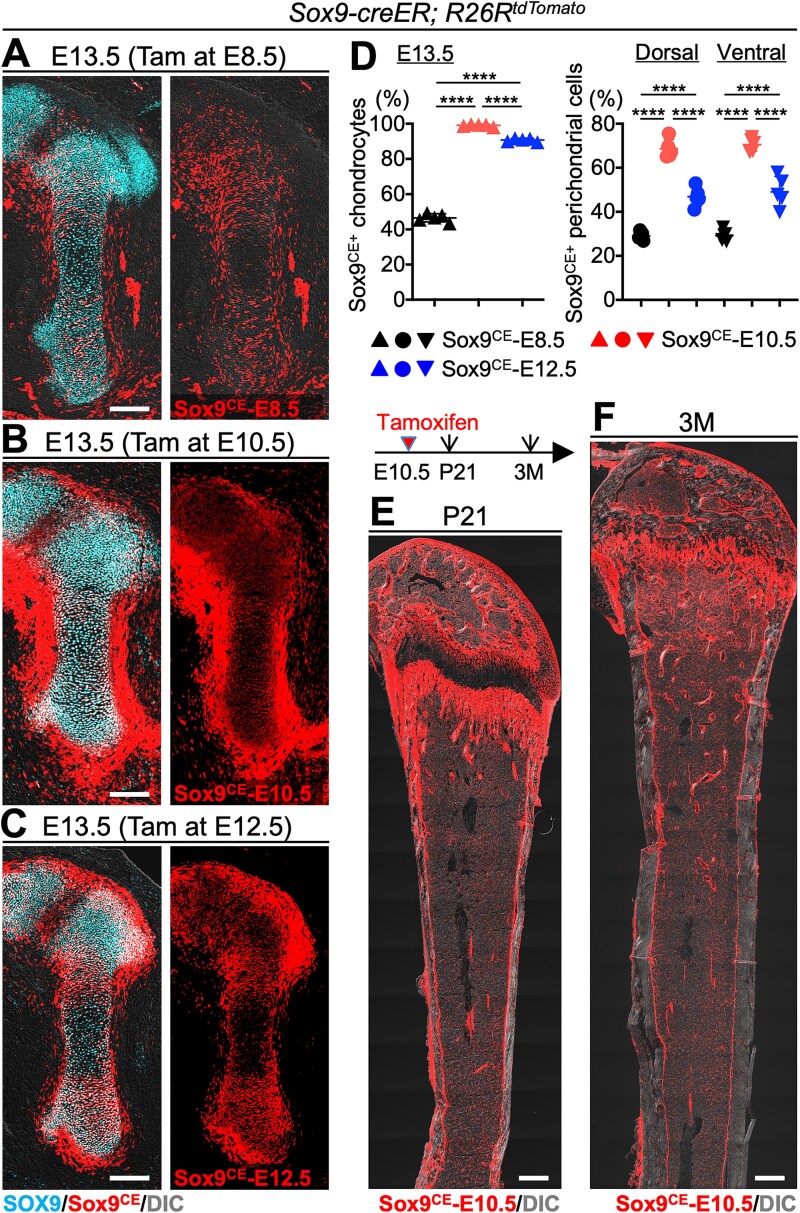
The contribution of spatiotemporally specific embryonic *Sox9-creER*-targeted cells to long-bone development. (A-C) SOX9 immunostaining of *Sox9*^CE^-tomato^+^ cells in the limb buds of *Sox9-creER*; *R26R*^tdTomato^ mice induced with tamoxifen at E8.5 (A), E10.5 (B), and E12.5 (C), and analyzed at E13.5. scale bar: 200 μm. (D) Quantitative analysis of *Sox9*^CE^-tomato^+^ cells in cartilage (left) and perichondrium (right) at E13.5. The proportion of *Sox9*^CE^-tomato^+^ cells in both areas increases from E8.5 to E10.5 and declines by E12.5. *n* = 5 mice per each group. ^****^p < .0001. Two-tailed, One-way ANOVA followed by Tukey’s post hoc test. Data are presented as mean ± SD. (E, F) *Sox9*^CE^-tomato^+^ cells in the long bones of mice induced at E10.5 and analyzed at P21 (E) and 3M (F). Scale bar: 500 μm. *Sox9*^CE^-tomato^+^ cells persist in the growth plate cartilage and cortical bone at both stages, demonstrating their long-term contribution to skeletal development.

Subsequently, we traced the fate of *Sox9*-expressing cells during bone development. *Sox9-creER*; *R26R*^tdTomato^ mice were treated with tamoxifen at E8.5, 10.5, or 12.5, and their femurs were analyzed at E13.5. We evaluated the distinct lineages of early *Sox9-creER* (*Sox9*^CE^)^+^ cells using SOX9 immunostaining (Figure 2A-C). Immunostaining at E13.5 revealed that the proportion of *Sox9*^CE^-tdTomato^+^ cells within the cartilage and perichondrium varied significantly depending on tamoxifen administration time.

*Sox9* ^CE^-tdTomato^+^ cells induced at E8.5 (*Sox9*^CE^-E8.5 cells) were detected in the cartilage and surrounding perichondrium at a lower proportion (Figure 2A). Quantitatively, these cells accounted for less than 50% of the chondrocytes and a much smaller percentage in the perichondrium (Figure 2D). *Sox9*^CE^-E10.5 cells showed nearly complete overlap with SOX9^+^ chondrocytes (Figure 2B, D). In the perichondrium, lineage cells of comprised approximately 70% of the perichondrial cells, highlighting the significant contribution of Sox9-expressing progenitors at this stage (Figure 2D). *Sox9*^CE^-E12.5 cells accounted for 90% of chondrocytes and 50% of perichondrial cells (Figure 2C, D). These results indicate that E10.5 represents a critical time-point for *Sox9*-expressing progenitors that contribute to the cartilage and perichondrium. Notably, the lineage of *Sox9*^CE^-tdTomato^+^ cells showed no differences between the dorsal and ventral sides at any time-point.

To evaluate the long-term contribution of *Sox9*^CE^-E10.5 cells, we analyzed femurs at P21 and 3 months (3M). *Sox9*^CE^-E10.5 cells were abundantly distributed among all skeletal cells at P21 (Figure 2E). These descendant cells remained prominently localized in the skeletal tissue, demonstrating their sustained contribution to maintenance and growth of long bones (Figure 2F).

The dynamic localization and proportion of *Sox9*^CE^-tdTomato^+^ cells revealed the distinct temporal roles of *Sox9*-expressing progenitors in skeletal development. Importantly, these *Sox9*^+^ cells contributed universally to the skeletal elements in the early skeletal developmental stage. *Sox9*^+^ cells at E10.5 exhibited peak contributions to cartilage and perichondrium, with long-term persistence in postnatal skeletal structures, suggesting they are potentially robust osteochondroprogenitors. These findings highlight the critical role of *Sox9*^+^ progenitors in driving lineage specification and structural development in long bones.

### Early *Fgfr3-creER*-targeted subsets of condensation contribute to dorsal skeletal components

**Figure 3:**
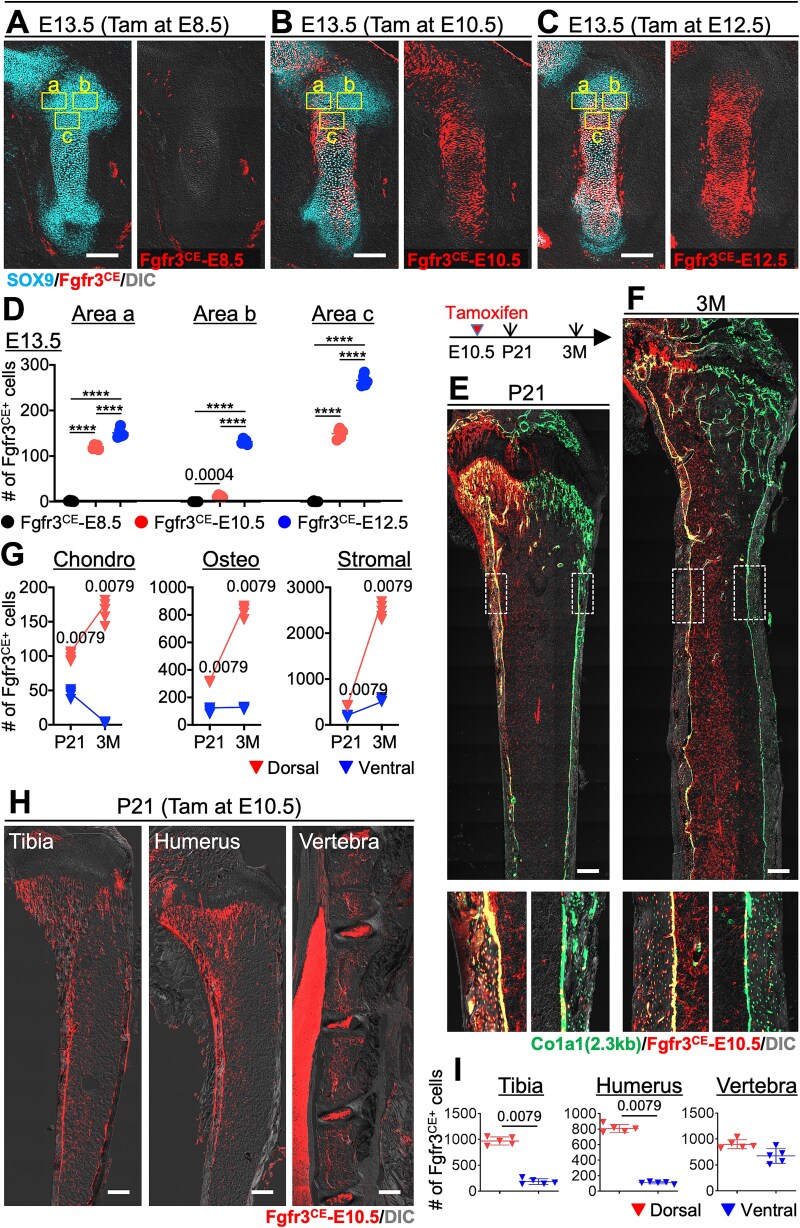
Early *Fgfr3-creER*-targeted subsets of condensation contribute to the dorsal skeletal components. (A-C) Immunostaining images for SOX9 in the cartilage anlage of *Fgfr3-creER*; *R26R*^tdTomato^ mice at E13.5. Tamoxifen was administered at E8.5 (A), E10.5 (B), or E12.5 (C), and femurs were analyzed at E13.5. Area a: dorsal resting zone, Area b: ventral resting zone, and Area c: proliferating zone. Scale bar: 200 μm. (D) Quantitative analysis of the number of *Fgfr3*^CE^-tomato^+^ cells in Areas a (dorsal resting zone), b (ventral resting zone), and c (proliferating zone) at E13.5. *n* = 5 mice per each group. ^****^p < .0001. Two-tailed, One-way ANOVA followed by Tukey’s post hoc test. Data are presented as mean ± SD. (E, F) Long-term contribution of *Fgfr3*^CE^-tomato^+^ cells at P21 (E) and 3M (F) pulsed at E10.5. Scale bar: 500 μm. (G) Quantitative analysis of the number of *Fgfr3*^CE^-tomato^+^ cells in chondrocytes, osteoblasts, and stromal cells at P21 and 3M. *n* = 5 mice per each group. Two-tailed, Mann-Whitney *U* test. Data are presented as mean ± SD. (H) Representative immunofluorescence images of P21 tibia, humerus, and vertebra from *Fgfr3-creER; R26R^tdTomato^* mice pulsed with tamoxifen at E10.5. Red: tdTomato^+^  *Fgfr3*-lineage cells. Scale bars: 500 μm. (I) Quantification of tdTomato^+^  *Fgfr3*^CE^-E10.5 cells in dorsal and ventral compartments of tibia (left) and humerus (right). *n* = 5 mice per each group. Two-tailed, Mann-Whitney *U* test. Data are presented as mean ± SD.

We investigated the spatiotemporal contributions of *Fgfr3*-expressing cells to skeletal development. *Fgfr3-creER*; *R26R*^tdTomato^ mice were treated with tamoxifen at E8.5, E10.5, or E12.5. We have previously reported the cell lineage at E12.5; in this study, we focused on an earlier stage. The femurs were analyzed at E13.5, using SOX9 immunostaining (Figure 3A-C). Compared with *Sox9*^CE^-lineage cells, *Fgfr3*-creER (*Fgfr3*^CE^)-E8.5 cells were undetected in the cartilage and perichondrium at E13.5. In contrast, *Fgfr3*^CE^-E10.5 and *Fgfr3*^CE^-E12.5 cells were distributed to most of the hypertrophic chondrocytes, some resting and proliferating chondrocytes marked by *Sox9*, and a few perichondrial cells, suggesting that *Fgfr3* expression begins in the limb bud around E10.5, following *Sox9*. *Fgfr3*^CE^-E12.5 cells seemingly contributed more to the resting and proliferating zones as compared to *Fgfr3*^CE^-E10.5 cells. To clarify the differences among these 3 groups, we established 3 distinct areas (a: dorsal resting zone, b: ventral resting zone, and c: proliferating zone) and quantified the contribution of stage-specific *Fgfr3*^CE^ lineage cells (Figure 3D). Consistent with the histological analysis, *Fgfr3*^CE^-E8.5 cells did not contribute to any area (Figure 3A, D). In Area a, *Fgfr3*^CE^-E10.5 and *Fgfr3*^CE^-E12.5 cells contributed in a similar manner. *Fgfr3*^CE^-E12.5 cells significantly contributed to Areas b and c as compared with the others, although moderate numbers of *Fgfr3*^CE^-E10.5 cells were found in Area c. Notably, no *Fgfr3*^CE^-E10.5 cells were detected in Area b (Figure 3B-D). The temporal dynamics of *Fgfr3*^+^ lineage cells during the embryonic stages revealed a progressive expansion of *Fgfr3*-expressing progenitors into additional regions of condensation, while maintaining a dorsal bias. These findings indicate that *Fgfr3*^CE^-tdTomato^+^ cells, labeled during the condensation stage at E10.5, specifically contribute to the dorsal skeletal components. Notably, the condensation stage represents a critical period for the specification and initial distribution of progenitors.

To assess the long-term contribution of *Fgfr3*^CE^-tdTomato^+^ cells, *Col1a1(2.3 kb)-GFP*; *Fgfr3-creER*; *R26R*^tdTomato^ mice were injected with tamoxifen at E10.5, and their femurs were analyzed at P21 and 3M (Figure 3E-G). Remarkably, *Fgfr3*^CE^-E10.5 cells were predominantly observed in chondrocytes, osteoblasts, and bone marrow stromal cells only within the dorsal metaphyseal area at P21 and 3M (Figure 3E, F). Quantitative analysis revealed a continuous increase in *Fgfr3*^CE^-E10.5 cells on the dorsal side during this period. This consistent contribution to the dorsal metaphysis aligns with their embryonic distribution at E13.5 and highlights the durable role of *Fgfr3*-expressing progenitors in skeletal maintenance and growth.

We next determined whether the dorsal-restricted contribution of *Fgfr3*^CE^-E10.5 cells is conserved beyond the femur. We extended our lineage-tracing analysis to additional skeletal elements, including the tibia, humerus, and vertebrae. In both the tibia and humerus, *Fgfr3*^CE^-E10.5 cells exhibited dorsal-restricted contributions to postnatal skeletal components, recapitulating the pattern observed in the femur. At P21, tdTomato^+^ cells were predominantly localized to the dorsal metaphyseal regions of the growth plate, cortical bone, and bone marrow stroma (Figure 3E, H). Quantitative analysis confirmed that the majority of *Fgfr3*^CE^-E10.5 cells resided in dorsal compartments, supporting a conserved dorsoventral bias across appendicular skeletal elements (Figure 3I). In contrast, the vertebral column did not show a clear dorsoventral restriction. *Fgfr3*^CE^-E10.5 cells were generally distributed across both dorsal and ventral regions of the vertebral body without significant differences in cell density at P21 (Figure 3H, I). However, we observed that in some vertebral segments, *Fgfr3*^+^ descendants appeared to be locally enriched on the dorsal side. These findings indicate that dorsoventral patterning of *Fgfr3*^CE^-E10.5 cells is robust and conserved in at least appendicular bones.

To address whether ventral counterparts to dorsal *Fgfr3*^+^ progenitors exist, we analyzed a published dataset (GSE126966) of the cartilage anlage at E13.5 from *Col2a1-cre*; *R26R*^tdTomato^ mice, focusing on tdTomato^+^ cells. Clustering analysis revealed 8 mesenchymal populations (Figure S1A, B). Among these, 2 candidate genes, *Tbx18* and *Tnc*, were identified as potential ventral markers based on their expression at the border zone of Fgfr3 expression within the Sox9-positive region (Figure S1C, D). To validate these findings, we performed immunofluorescence on E13.5 cartilage anlage. TBX18 and TNC proteins were localized exclusively to the ventral side (Figure S1E). We therefore propose Tbx18 and Tnc as candidate markers for early ventral progenitors in the cartilage anlage.

The spatial restriction of *Fgfr3*^CE^-tdTomato^+^ cells to the dorsal regions during both the embryonic and postnatal stages underscores their lineage-specific contribution to skeletal development. The predominance of stromal cells among postnatal *Fgfr3*^CE+^ populations suggests that *Fgfr3*-expressing progenitors play a critical role in dorsal metaphyseal skeletal components and contribute to establishing the bone marrow microenvironment.

### Early *Dlx5-creER*-targeted perichondrial subsets contribute to the dorsal diaphyseal skeletal components

**Figure 4:**
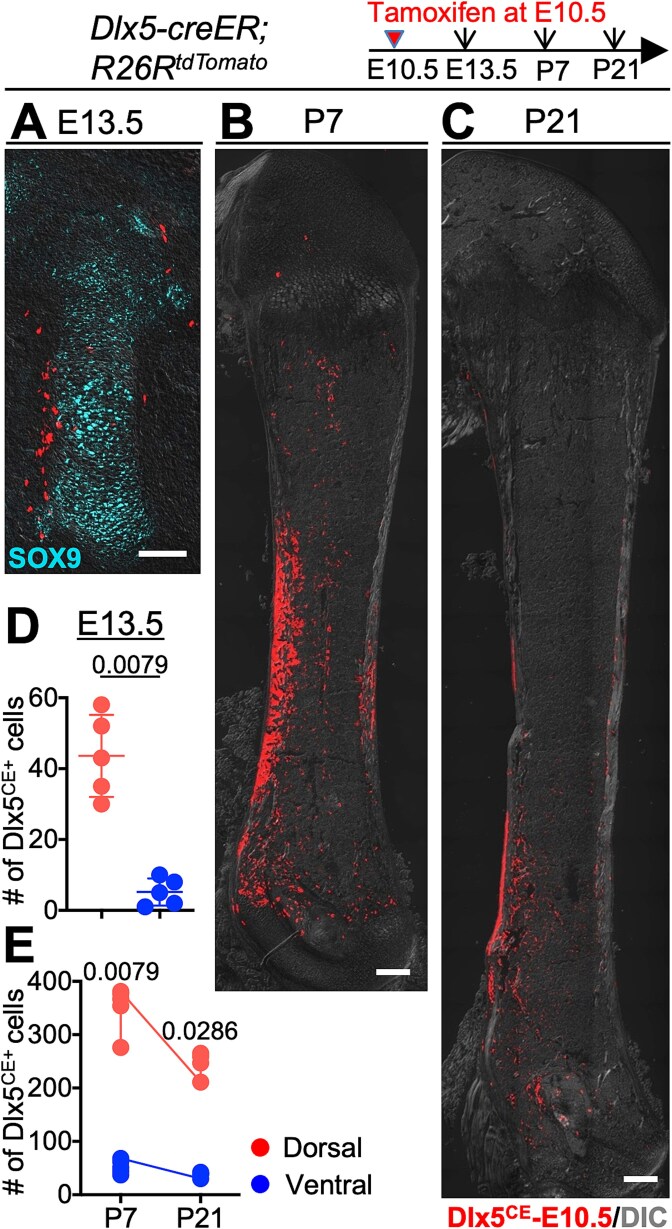
Early *Dlx5-creER*-targeted perichondrial subsets contribute to the dorsal diaphyseal skeletal component. (A-C) Cell lineage of *Dlx5*^CE^-tomato^+^ cells from *Dlx5-creER*; *R26R*^tdTomato^ mice pulsed at E10.5. Femurs at E13.5 (A), P7 (B), and P21 (C). Scale bar: 200 μm (A) and 500 μm (B, C). (D, E) Quantitative analysis of the number of *Dlx5*^CE^-tomato^+^ cells in dorsal and ventral sides at E13.5 (D), P7, and P21 (E). *n* = 4 (P21) or 5 (E13.5 and P7) mice. Two-tailed, Mann-Whitney *U* test. Data are presented as mean ± SD.

*Dlx5* is expressed in the outer layers of both dorsal and ventral perichondrial cells during the cartilage anlage stage.^9^ Although scRNA-seq results showed minimal expression of *Dlx5* at E10.5 (Figure 1C, D), we administered *Dlx5-creER*; *R26R*^tdTomato^ mice with tamoxifen at E10.5 to elucidate the precise skeletal landscape. The femurs were analyzed at E13.5, P7, and P21 to assess the spatiotemporal contribution of *Dlx5*^CE^-tdTomato^+^ cells to the dorsal and ventral skeletal components. Overall, a few *Dlx5*^CE^-E10.5 cells were located in the perichondrium; however, they were predominantly localized to the dorsal area, with significantly fewer cells observed on the ventral side (Figure 4A, D). This dorsal-biased distribution highlights the early role of *Dlx5*-expressing perichondrial cells in dorsal skeletal components. *Dlx5*^CE^-E10.5 cells remained primarily in the dorsal diaphyseal area, with significantly higher numbers than those on the ventral side at P7 (Figure 4B, E). These cells were distributed within the diaphyseal cortical bone and adjacent skeletal structures, demonstrating their specific contribution to dorsal skeletal elements. These cells continued to exhibit strong diaphyseal dorsal localization until P21, although their numbers were slightly reduced compared to those at P7 (Figure 4C, E).

These findings demonstrate that *Dlx5*^CE^-tdTomato^+^ cells in the condensation stage contribute specifically and durably to the dorsal diaphyseal skeletal components. Their dorsal-restricted distribution and long-term persistence underscore the critical roles of *Dlx5*-expressing perichondrial subsets in skeletal patterning and maintenance.

### *Fgfr3-creER* cells in condensation functionally important in bone development

**Figure 5:**
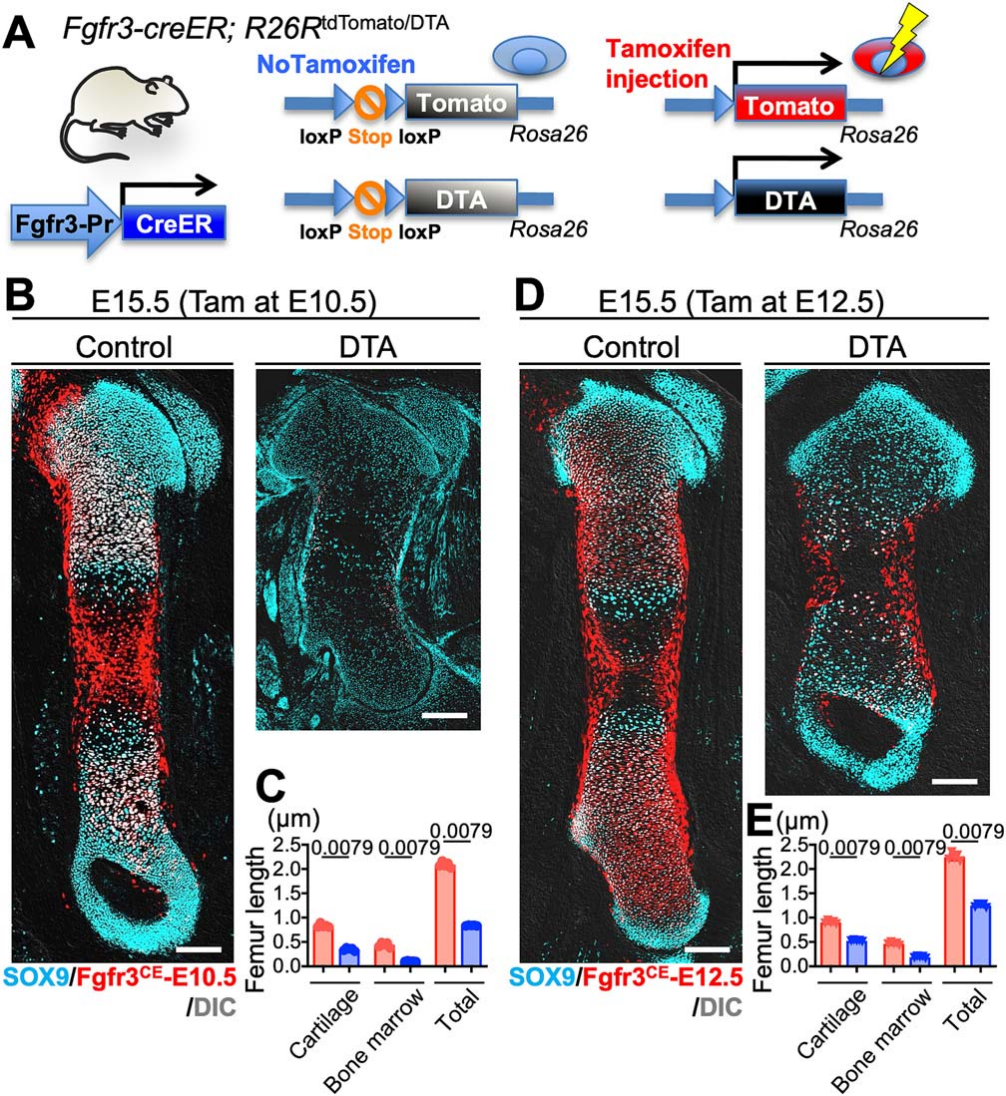
*Fgfr3-creER* cells in condensation are functionally important in bone development. (A) Overview of *Fgfr3-creER*; *R26R*^tdTomato/DTA^ system. tdTomato expression and DTA induction concomitantly occur. (B) Representative images of long bone from *Fgfr3-creER*; *R26R*^tdTomato/iDTA^ (DTA) and *Fgfr3-creER*; *R26R*^tdTomato/+^ (Control) mice pulsed at E10.5. Scale bar: 200 μm. (C) Quantification of the length of cartilage, bone marrow cavity, and whole bone at E15.5 in Control and DTA mice. *n* = 5 mice per each group. Two-tailed, Mann-Whitney *U* test. Data are presented as mean ± SD. (D) Representative images of long bone from *Fgfr3-creER*; *R26R*^tdTomato/iDTA^ (DTA) and *Fgfr3-creER*; *R26R*^tdTomato/+^ (Control) mice pulsed at E12.5. Scale bar: 200 μm. (E) Quantification of the length of cartilage, bone marrow cavity, and whole bone at E15.5 in Control and DTA mice. Control (red) and DTA (blue) groups. *n* = 5 mice per each group. Two-tailed, Mann-Whitney *U* test. Data are presented as mean ± SD.

Lastly, to evaluate the functional significance of *Fgfr3*^+^ cells in embryonic bone development, we performed inducible cell ablation experiments using a *Rosa26*^iDTA^ (inducible diphtheria toxin fragment A) allele.^23^ By administering tamoxifen to *Fgfr3-creER*; *R26R*^tdTomato/iDTA^ mice at E10.5 or E12.5, *Fgfr3*^+^ cells began to express tdTomato and cell death was simultaneously induced (Figure 5A). The femurs were assessed at E15.5, by comparing *Fgfr3-creER*; *R26R*^tdTomato/+^ (control) and *Fgfr3-creER*; *R26R*^tdTomato/iDTA^ (DTA) littermates. In the groups administered with tamoxifen at E10.5 (*Fgfr3*^CE^-E10.5-DTA), a drastic reduction in the number of *Fgfr3*^CE^-tdTomato^+^ cells and their progeny was observed. Notably, a significant impairment in bone development was observed in the *Fgfr3*^CE^-E10.5-DTA group compared to the control group (Figure 5B). Femur length, including cartilage, bone marrow cavity, and total bone lengths, was significantly shorter in *Fgfr3*^CE^-E10.5-DTA mice than in controls (Figure 5C). Control femurs exhibited well-organized bone structures with robust cartilage and bone marrow cavity development. *Fgfr3*^CE^-E12.5-DTA also showed developmental deficiency, but was less pronounced than *Fgfr3*^CE^-E10.5-DTA (Figure 5D, E). tdTomato^+^ cells were still observed in the DTA at this stage; however, tdTomato^+^ cells from E10.5 were not, probably because of the shorter recombination duration.

These findings emphasize the critical role of *Fgfr3*^+^ cells in initiating and supporting early bone development during the early embryonic stages.

## Discussion

**Figure 6:**
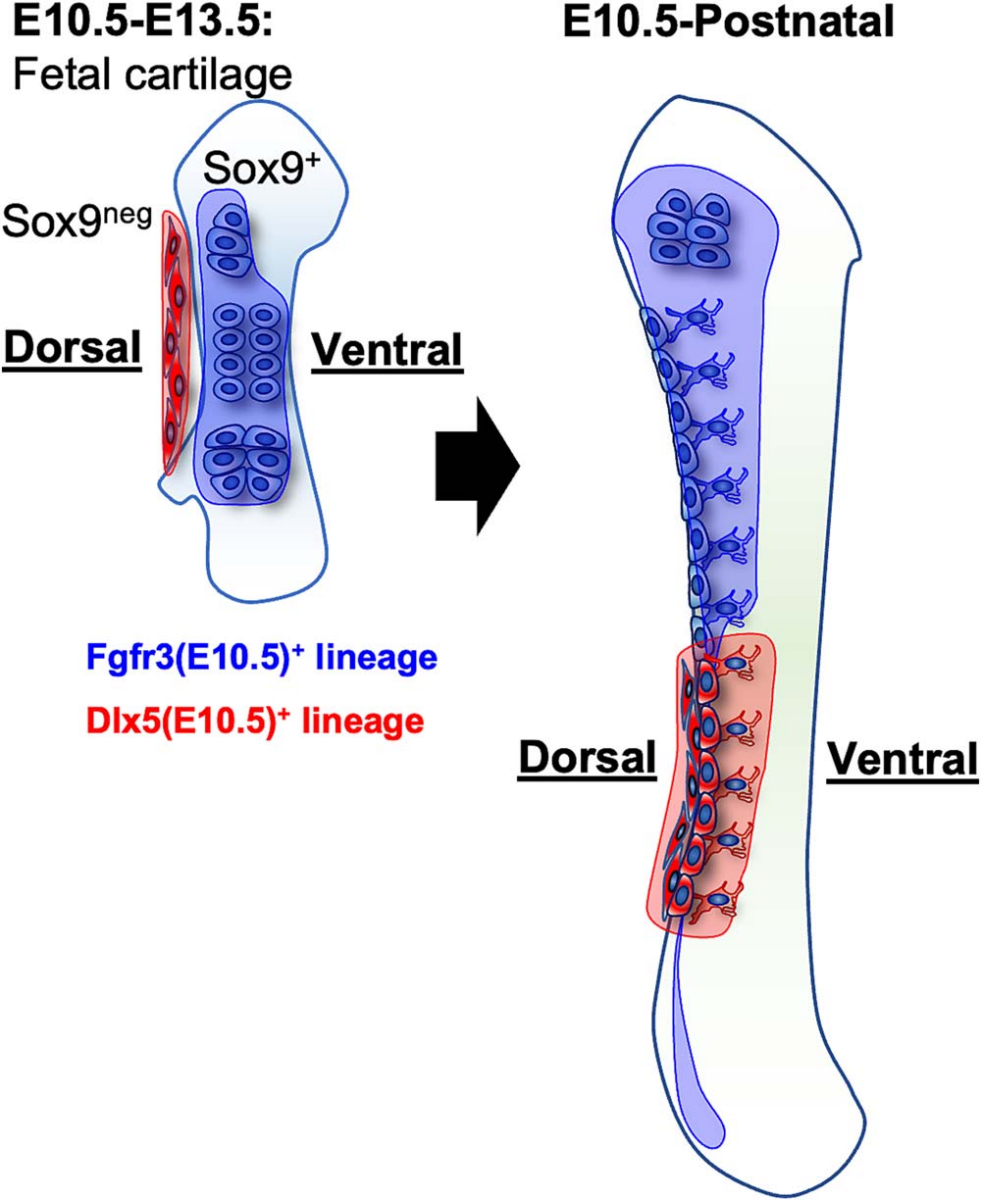
Early determination of the dorsal-ventral axis in long-bone development. The condensation stage represents a pivotal period for the formation of skeletal progenitors and the establishment of dorsal-ventral patterning during bone development. *Fgfr3* begins to express at approximately E10.5. These *Fgfr3*^+^ cells contribute to dorsal resting and proliferating chondrocytes as well as all hypertrophic chondrocytes. Ultimately, their lineage cells are exclusively distributed on the dorsal metaphyseal area of long bones in the adult stage.

This study highlights the critical significance of the mesenchymal condensation stage as a developmental window for skeletal progenitor cells, and underscores the distinct contributions of dorsal and ventral progenitor populations to skeletal development. By investigating the *Sox9*^+^, *Fgfr3*^+^, and *Dlx5*^+^ subsets, we demonstrated the contributions of these progenitors to the spatiotemporal dynamics required for skeletal development (Figure 6).

*Sox9-creER*-targeted cells at E10.5, contribute nearly universally to the cartilage and perichondrium, helping establish the foundational cellular framework for long-bone development.^4^ In contrast, *Fgfr3*^+^ progenitors exhibit a pronounced dorsal-specific contribution to skeletal components, with E10.5-ablation resulting in severe disruption of long-bone growth and structural integrity. Similarly, *Dlx5*^+^ cells emerged as key players at E10.5, contributing exclusively to dorsal skeletal structures. Their dorsal-restricted localization and long-term persistence suggest that *Fgfr3*^+^ and *Dlx5*^+^ progenitors uniquely shape the dorsal cortical bone and the diaphyseal structure. Together, these findings highlight the irreplaceable contributions of E10.5 progenitors in driving skeletal specification and establishing lineage-specific developmental trajectories. This conclusion was also briefly mentioned in a study by Yu et al., who observed that signaling pathways regulating the commitment of mesenchymal cells to chondrogenic or osteogenic lineages peaked at E10.5.^24^ In contrast, our research focused on the spatial distribution of specific progenitor cell types and provided a functional validation of their roles. The long-term persistence of *Sox9*^+^, *Fgfr3*^+^, and *Dlx5*^+^ progenitors into postnatal life suggests their broader roles in skeletal biology. These cells contribute to the growth plate, cortical bone, and stromal compartments, underscoring their ongoing involvement in bone maintenance and remodeling. These results align with those of previous studies, suggesting that progenitor cells play a critical role during the development and maintenance of adult bone, particularly in growth plate cartilage and bone marrow stroma.^25–27^ In our study, lineage-tracing techniques provided deeper insight into the specific origins and spatial distributions of these progenitor cells. Moreover, identifying lineage-specific contributions provides a foundation for developing targeted therapies. For example, upregulation of *Sox9* may enhance cartilage repair, whereas modulating the *Fgfr3* and *Dlx5* pathways could address defects in the cortical bone or dorsal skeletal elements.

Compared to earlier and later stages, such as E8.5 or E12.5, E10.5 emerges as a decisive stage where *Sox9*^+^, *Fgfr3*^+^, and *Dlx5*^+^ progenitors established distinct developmental programs. Our study demonstrated that at E8.5, mesenchymal condensation remained in its early preparatory phase. During this early period, progenitor cells reportedly exhibit minimal expression of chondrogenic and osteogenic markers, such as *Sox9*, *Prrx1*, and *Col2a1*, supporting the present study findings.^28–30^ The E10.5 stage was marked by aggregation and proliferation without significant lineage commitment. Research has shown that at E12.5, the mesenchymal progenitor cells enter a transitional phase, shifting from a multipotent state to specific lineage roles.^31^ During this stage, progenitor cells begin to assume more defined roles within the condensation zones, marking an important step in the progression toward fully specialized skeletal development.^9^^,^^32^ Chondroprogenitors exhibit a distinct zonal organization, characterized by enhanced chondrogenic activity,^33^ whereas osteoprogenitors express key markers, such as *Sp7 (Osx)* and *Runx2.*^34^ Our *iDTA*-induced cell ablation experiments reinforced this temporal specificity, as disruptions at E10.5 led to severe structural defects, whereas disruptions at E12.5 resulted in milder abnormalities. Other studies have also mentioned the importance of E10.5, which stems from it being a critical stage during which progenitor cells respond to signaling, such as BMP, FGF, and WNT, to establish the cellular framework essential for skeletal growth.^35–37^

The spatial distinction between the dorsal and ventral contributions further emphasizes the complexity of skeletal development. *Fgfr3*^+^- and *Dlx5*^+^-descendant cells derived at E10.5 predominantly contribute to the dorsal regions. These results align with earlier zebrafish study findings, where *Dlx* expression followed the dorsal-ventral axis, particularly during craniofacial skeletal formation.^38^^,^^39^ These findings suggest that this dorsal-ventral axis is conserved in vertebrates and likely plays a central role in the skeletal development in mice.^39–41^

These findings also have implications for understanding congenital skeletal disorders. Disruptions in the activities of *Sox9*^+^, *Fgfr3*^+^, or *Dlx5*^+^ progenitors at E10.5 may underlie osteogenic conditions such as scoliosis or long-bone deformities. Furthermore, the interaction between the BMP, FGF, and WNT signaling pathways likely helps establish progenitor-specific trajectories, and understanding these interactions could reveal new therapeutic targets. Our findings may possess therapeutic potential, particularly for conditions such as achondroplasia resulting from activating mutations in FGFR3. Building upon our previous study^9^ and the current research, we have delineated the temporal expression pattern and lineage contribution of *Fgfr3*^+^ cells in skeletal development. These studies collectively elucidate the developmental trajectory and structural contribution of Fgfr3-expressing progenitors. While significant ethical considerations remain, our results provide a rationale for investigating time-specific pharmacological interventions or cell-based therapies, including during fetal stages, as potential strategies to modulate FGFR3-related skeletal disorders. Future studies could benefit from combining lineage tracing with single-cell transcriptomics to explore how these signaling pathways drive progenitor activity during this critical window. Comparative studies across species may also shed light on the evolutionary mechanisms underlying dorsoventral asymmetry and skeletal complexity.

The limitations of this study include the fact that recombination induced by the CreER driver may not occur with complete specificity and that it is not possible to continuously track the lineage within a single individual. However, to address these limitations, we used multiple samples to achieve statistical significance and employed multiple mouse lines to enhance the reliability of our findings.

Focusing on the initial condensation stage, our study clarified how progenitors shape skeletal development and patterning. The distinct contributions of *Sox9*^+^, *Fgfr3*^+^, and *Dlx5*^+^ progenitors, along with the spatiotemporal aspects of their activity, offer new avenues for understanding skeletal biology and designing interventions for skeletal disorders and regenerative medicine.

## Supplementary Material

FigS1_zjaf086

## Data Availability

Source data are provided with this paper. The raw data generated during and/or analyzed during the current study are available from the corresponding author upon reasonable request. The single-cell RNA-seq data presented herein (GSE144411 and GSE126966) were downloaded from the GEO database. Other source data for figures in the paper is available from the Lead Contact upon reasonable request.
