# Supplementary figures and images for: Early determination of the dorsal-ventral axis in endochondral ossification in mice
Source: J Bone Miner Res. 2025 Jun 23;40(12):1385–96. doi: 10.1093/jbmr/zjaf086 (PMC12685720; doi:10.1093/jbmr/zjaf086)

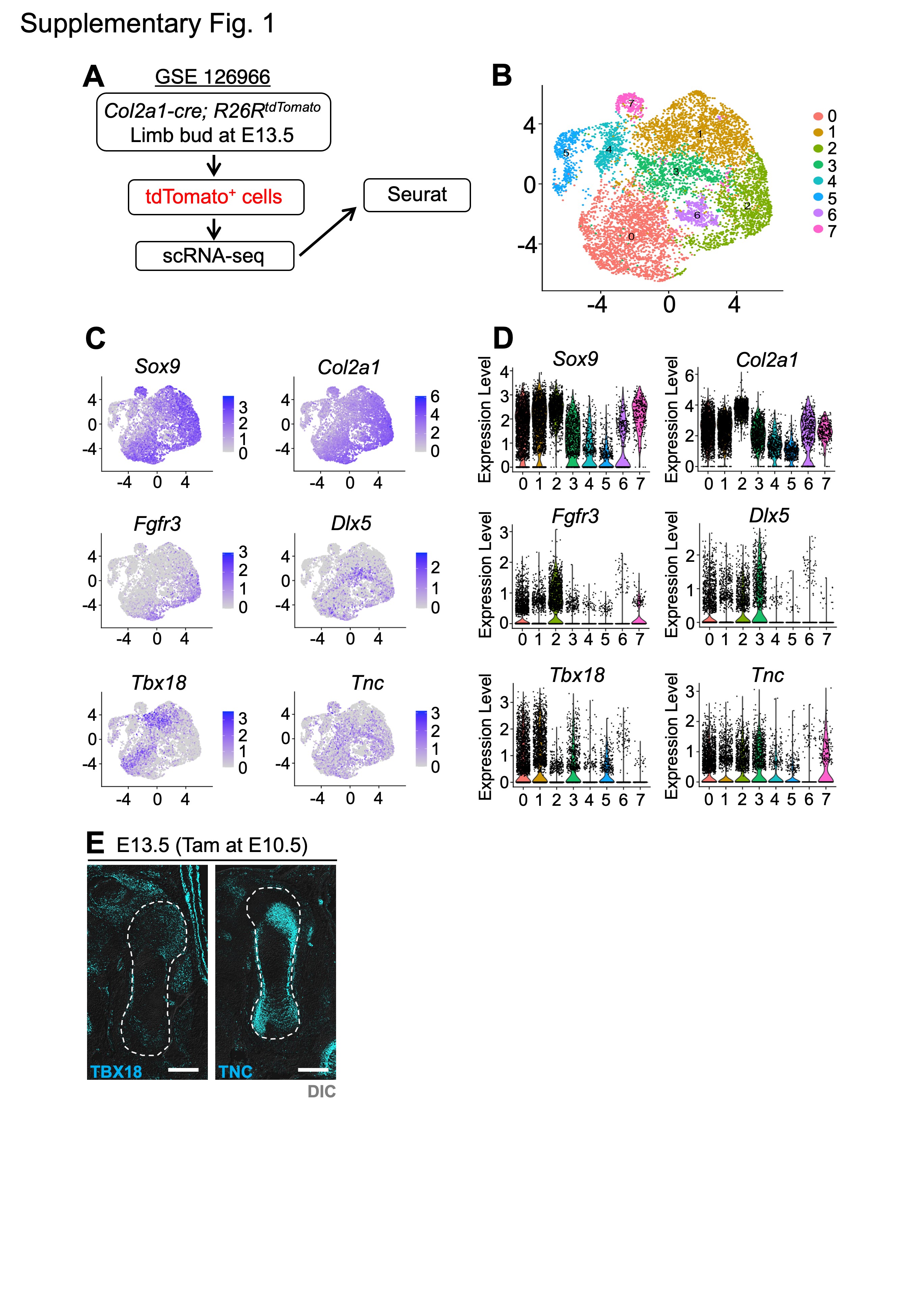

Supplement: FigS1_zjaf086 [file figs1_zjaf086.jpeg]
